# Compliance of commercial motorcycle riders with road safety regulations in a peri-urban town of Ghana

**DOI:** 10.1371/journal.pone.0246965

**Published:** 2021-03-30

**Authors:** Dorcas Hagan, Elvis E. Tarkang, Fortress Yayra Aku

**Affiliations:** 1 Department of Epidemiology and Biostatistics, School of Public Health, University of Health and Allied Sciences, Hohoe, Ghana; 2 Department of Population and Behavioural Sciences, School of Public Health, University of Health and Allied Sciences, Hohoe, Ghana; Tongii University, CHINA

## Abstract

**Background:**

While motorcycles are essential for moving people and goods, they are also, a significant contributor to Road Traffic Accidents (RTAs), making it a public health issue of concern globally. The Hohoe Municipal Hospital records increasing RTAs due to commercial motorcycles. Determining motorcycle riders’ compliance with road safety regulations is critical in helping to curb this menace.

**Method:**

A cross-sectional study was employed involving a multistage sample of 238 motorcycle riders. Data were collected using a pretested structured questionnaire and entered into Epi Data version 3.2 software and exported to STATA software version 12 for analysis. Descriptive and inferential analyses were done while statistical significance was determined at 95% reliability interval and p-value of 0.05.

**Findings:**

The level of compliance with road safety regulations among respondents was 59.2%. The mean age of respondents was 29.9 ± 7.9 years, and all respondents were males. Respondents who did not own their motorbikes were 0.39 times less likely to comply with road safety regulations compared to their counterparts who owned one, while those without alternate occupations were 0.51times less likely to comply with road safety regulations compared to those with an additional occupation. Those aged between 30–39 years and 40–49 years were 2.37 and 4.1 times more likely to comply with road safety regulations, respectively, compared to those aged ≤29 years, and those who did not smoke were 3.15 times more likely to comply with road safety regulations than those who smoked.

**Conclusion:**

Compliance to safety regulations are fairly low and although motorcycle usage on a commercial basis is yet to be legalised in Ghana, routine education targeting riders who smoke, do not have alternate occupations, do not own motorbikes and younger riders will improve their compliance. Also, riders should be encouraged to obtain their license from the appropriate authorities.

## Introduction

Although motorcycles are essential for moving people and goods, they are also a significant contributor to Road Traffic Accidents (RTAs), resulting in injuries and fatalities [1]; hence, it is a public health issue of concern globally. RTAs are currently ranked the ninth leading cause of death for all age groups globally and are even projected to become the seventh leading cause of death by 2030 [2]. It has been proven that most motorcycle related fatalities (90%) occur in low and middle-income countries, which is attributed to their huge motorcycles’ usage [2].

Studies show that Africa is the least mobile continent in terms of vehicular movement. The continent has the lowest number of vehicles, representing 2 percent of the world’s vehicle fleets [3]. However, it is currently rapidly experiencing an increase in commercial motorcycle usage. Consequently, the highest recorded RTA-related mortality rate in the world occurs there, with passengers and pedestrians being the most affected [4, 5].

According to statistics from the Ghana Motor Traffic and Transport Department, a total of 2,076 persons perished in motorcycle traffic accidents in 2017, with 3,487 motorcycles being involved in these accidents. The data also shows that, out of the persons who died, a high proportion of 39.6% (823) died in the Volta region [6]. The World Health Organization (WHO) recommends the establishment and adherence to speed limits, road designs based on their functions, construction of elements of the road infrastructure, enforcement of speed limits, and use of safety helmets and seatbelts to improve compliance and reduce RTAs [2]. The emergence of a commercial motorcycle taxi business, popularly called “okada” in most Sub-Saharan African countries, including Ghana, was, to a larger extent, facilitated by a lack of proper laws and regulations. In addition to this worrying trend, the high non-compliance to road safety regulations have been reported among commercial motorcyclists. Though “okada” is not legal, it is imperative that commercial motorcyclists obey road safety regulations to safeguard their own lives and that of other road users. Safety road regulations for commercial motorcyclists are no different from the general road safety regulations for motorcyclists, which include crash helmet usage, use of protective clothing, usage of headlights while riding at night, provision of a crash helmet for passengers, no use of mobile phones and no alcohol or tobacco usage while working [7].

In Nigeria, a study found that 50.6% of its respondents comply with road safety regulations, while 25.6% had a good attitude towards road safety [8]. Also, out of 340 commercial motorcyclists studied in Uganda, only 0.9% of them complied to all four road safety measures investigated by the study, while 24.4% complied to at least three of the measures [7]. It is important to pay attention to the factors contributing to compliance among motorists and their consequences. Diverse factors such as the vehicle, driver, temporal, environmental, and roadway characteristics have been identified to contribute to compliance with road safety regulations and the severity of injury among truck drivers [9]. The same study also found that older vehicle, night-time, two-way traffic and nature of light during the day had a positive influence on injury severity for drivers involved in RTA. In another study, speed gap, length of a segment and wet road surfaces accounted for single and multi- vehicle accidents [10]. Therefore, tailored educational messages and robust driver training would improve compliance and subsequent reduction in road traffic accidents and injuries [11].

Reports from across Ghana illustrates how, in recognition of the shortcomings of enforcement of traffic regulations, speed bumps and rumble strips are sometimes installed by people in communities to reduce RTA related fatalities [12]. The commercial motorcycle business is increasingly being patronised in the Hohoe township due to the poor nature of roads linking some of the suburbs and communities. Records from the accident and emergency unit of the Hohoe Municipal Hospital show that commercial motorcycle-related RTAs in the Hohoe township are on the rise, resulting in severe injuries and mortality. This study, therefore, sought to determine the compliance of commercial motorcycle riders with road safety regulations and its association with their socio-demographic and individual-related characteristics.

## Materials and methods

### Study site

The study was conducted in Hohoe, the capital of the Hohoe Municipality, one of the 18 Districts/Municipalities in the Volta Region. The Municipality has a total population of 167,016 per the 2010 Population and Housing Census. It has seven sub-Municipalities, with health centres and Community-based Health Planning Service compounds and one municipal hospital, which serves as the referral facility for the Municipality. The Municipality has various means of transport with a substantial contribution from commercial motorcycles, due to the bad nature of most roads and hard-to-reach areas.

### Study design

A cross-sectional design was employed for this study involving 238 commercial motorcyclists recruited in the Hohoe township between February and March 2020.

#### Sample size determination

A compliance rate of 18% with road safety regulations among commercial motorcycle riders based on a study in Ethiopia [13] was used. The sample size was determined using the formula below:

$$n=\frac{Z_{\frac{a}{2}}^{2}p(1-p)}{d^{2}}$$

[14] Where

*n* = sample size required $Z_{\frac{a}{2}}^{2}$ = Z-Score = 1.96 at 95% Confidence interval *p* = proportion of study population = 18% *d*^2^ = margin of error With a margin of error of 5% Hence, the sample size $(n)=\frac{{\left(1.96\right)}^{2}\times 0.18\times (1-0.18)}{{\left(0.05\right)}^{2}}=226.80$ Addition of 5% non-response rate = 5%*226.80+226.80 = 238.

### Sampling

A multistage sampling method was used. Three commercial motorcycle operating clusters exist in the Hohoe township and were identified as cluster A, B and C for the purpose of this study. Proportionate sampling was then used to determine the number of motorcyclists to be recruited from each cluster. In that way, the total sample size was divided by the sampling frame obtained in each cluster and multiplied by 100 to obtain the proportion that was recruited per cluster. Finally, simple random sampling was used to select respondents. On pieces of papers, “Yes” or “No” was written, placed in a bowl and shaken. Data were collected thrice every week in February and March. On data collection days, motorcyclists were asked to pick from the bowl with the papers after every shake, those who picked “Yes” were recruited as respondents after giving consent.

### Data collection tool and procedure

Two trained research assistants collected data on compliance with road safety regulations and its associated factors (socio-demographic and individual-related) among respondents through interviews using a structured questionnaire. Prior to the data collection process, the questionnaire was pretested in the Kpando township, thus, allowing for identification of any gaps in the questionnaire to inform possible modifications. In addition, a Cronbach alpha test was run on the questionnaire, and 0.766 was obtained before the actual data collection.

### Data analysis

Data were entered into EpiData software version 3.1 and exported into Stata/MP 16.0 (Stata Corporation, College Station, Texas, USA) for analysis. Descriptive statistics were used to describe continuous and categorical variables. The primary unit of analysis, compliance to road safety regulation dichotomised as “0” for poor compliance, and “1” for good compliance was defined using seven (7) distinct variables. The variable “alcohol use while riding” was reverse coded, thus, “Yes” was coded 0 and “No” was coded 1. “Receive calls when riding” was also reverse coded as always/sometimes = 0 and Never/ Rarely = 1. Other variables, “wear a crash helmet” and “obeys traffic signs” were coded on a 3-point Likert scale; hence, 0 = never, 1 = sometimes/rarely and 2 = always. The seven variables yielded a minimum and maximum score of 1 and 10, respectively. Good compliance was defined as having a score of 7 and above, and poor compliance was defined otherwise. Multiple logistic regression was used to determine factors associated with compliance with road safety regulation. Variables with p<0.05 at 95% confidence interval were considered statistically significant. Clustering by locality was not accounted for in the statistical analysis. This is because clusters considered for this study are three locations (with no difference in characteristics) within the Hohoe township, where groups of commercial motorcycle riders normally gather to operate.

### Ethical issues

Approval for the study was obtained from the University of Health and Allied Science Research Ethics Committee with approval number UHAS-REC A.4 [80]19–20. Written permission was sought from the Hohoe Municipal Assembly and written consent sought from the respondents prior to data collection. All questionnaires were coded to avoid tracing information to respondents in ensuring anonymity. Additionally, questionnaires were kept safe under lock and key; they were accessible only to the research team to ensure confidentiality. Respondents were assured that they would not be reported to authorities on the grounds of non-compliance or whatsoever; hence, they were free to give their true responses. However, they were assured that findings from the study would aid stakeholders to engage and implement informed strategies to improve regulations compliance.

## Results

### Socio-demographic characteristics of respondents

Out of the 238 motorcycle riders who participated in the study, 76.9% owned motorbikes. The mean age of participants was 29.9 ± 7.9 years. Most (55%) were aged <29 years, with 65.2% being single at the time of the study. Over half (58.8%) of the respondents were Christians, whereas 60.9% had basic school education. Only 4.6% had tertiary level education, and a few (35.3%) resided in urban communities. Majority of the participants earned less than GHS 400 ($70.1) every week. About 20.6% and 61.3% were smokers and drinkers, respectively. Approximately 44.1% had other occupations aside from riding a motorbike, and 20.6% had been involved in a motor accident (Table 1).

**Table 1:** Socio-demographic characteristics of motor riders [n = 238].

Variables	Frequency	Percentage (%)
Mean ± SD = 29.9 ± 7.9		
***Background characteristics***		
***Age***		
<29	131	55.0
30–39	68	28.6
40–49	39	16.4
***Marital status***		
Married	83	34.8
Single	155	65.2
***Religion***		
Christian	140	58.8
Muslim	98	41.2
***Alternate occupation***	2	
Yes	105	44.1
No	133	55.9
***Level of education***		
No education	34	14.3
Basic education	145	60.9
Secondary	48	20.2
Tertiary	11	4.6
***Area of residence***		
Urban	84	35.3
Rural	154	64.7
***Weekly Income (GHC)***		
<400	235	98.7
>400	3	1.3
***Owner of a motorbike***		
Yes	183	76.9
No	55	23.1
***Current smoking status***		
Smoke	49	20.6
Does not smoke	189	79.4
***Current drinking status***		
Drinks	146	61.3
Does not drink	92	38.7
***Ever been involved in a motor accident***		
Involved	49	20.6
Never involved	189	79.4

#### Compliance to safety regulations indicators

Out of the 238 motorcycle riders, 53.8% had a licence to ride a motorbike. Out of the 95.8% of respondents who owned crash helmets, only 51.7% wore them often. Few (20.2%) of motor riders offered crash helmets to their passengers. Approximately 68.1% and 53.4% often wore protective clothing and consumed alcohol while riding, respectively. A majority (98.3%) used headlights while riding at night; however, only a few (39.5%) of them always obeyed traffic regulations (Table 2).

**Table 2:** Compliance to road safety guidelines [n = 238].

Variables	Frequency	Percentage (%)
***Licensed rider***		
Licensed	128	53.8
Not Licensed	110	46.2
***Owns a crash helmet***		
Owns	228	95.8
Does not own	10	4.2
†***Wears a crash helmet***		
Always	123	51.7
Sometimes	80	33.6
Never	35	14.7
†***Ensure passengers wear a crash helmet***		
Ensures	35	14.7
Does not ensure	203	85.3
†***Wears protective clothing***		
Wears	162	68.1
Does not wear	76	31.9
†***Uses alcohol while riding***		
Uses	127	53.4
Does not use	111	46.6
†***Uses headlights while riding at night***		
Uses	234	98.32
Does not use	4	1.68
†***Obeys traffic signs***		
Never/ Rarely	37	15.5
Sometimes	107	45.0
Always	94	39.5
† ***Receives calls when riding***		
Never/ Rarely	45	18.9
Sometimes	151	63.5
Always	42	17.7
***Frequency of motorbike service***		
Once every week	106	44.5
Once every month	127	53.4
Once every year	5	2.1
***Uses protective goggles when riding***		
Never/ Rarely	163	68.5
Sometimes	52	21.9
Always	23	9.7

Note:

^†^ Variables used to compute compliance to road safety regulations.

#### Distribution of compliance to safety regulations among motorbike and licence owners

**Fig 1:**
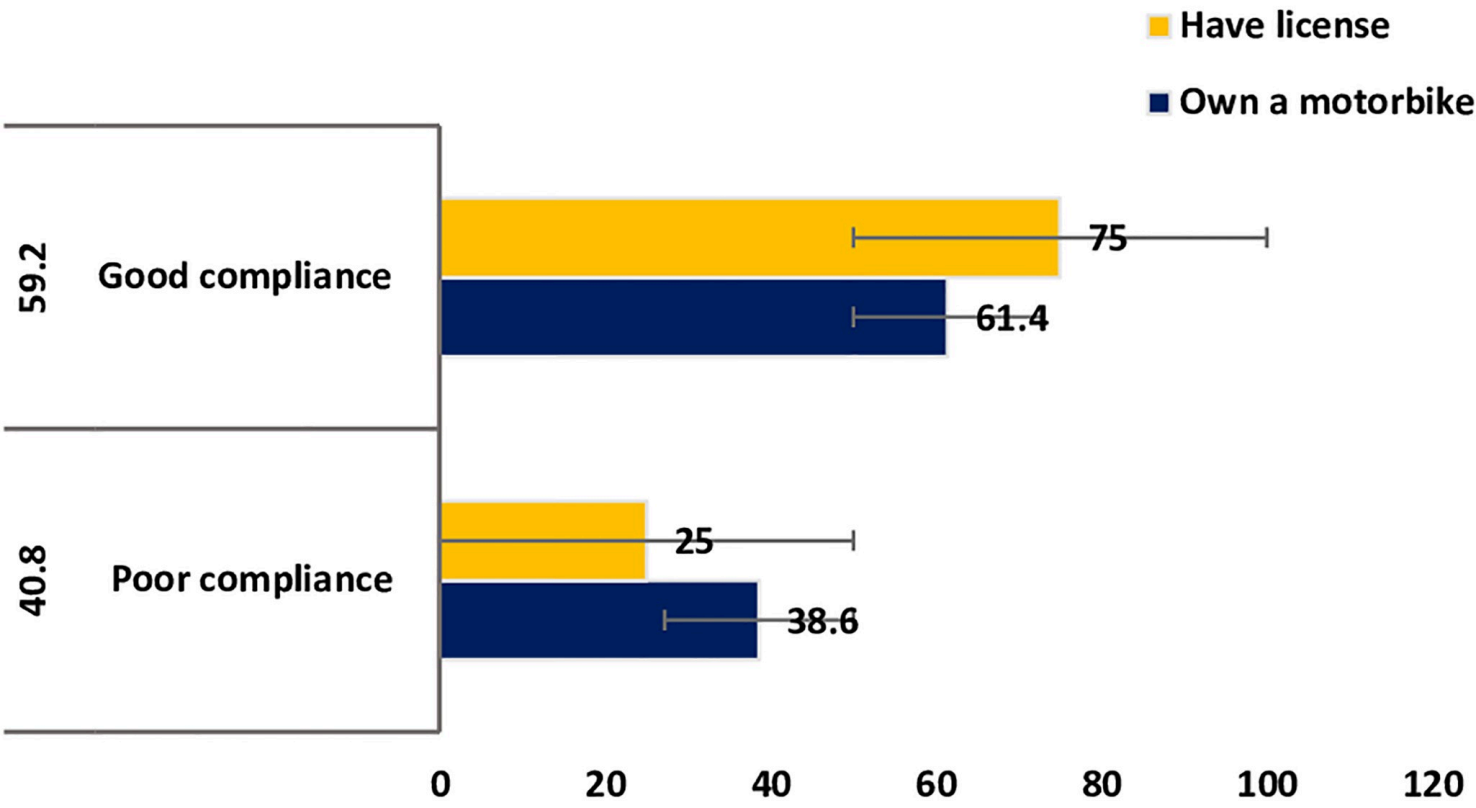
Compliance to road safety guidelines among motorbike and licence owners.

Good compliance with road safety regulations was prevalent among 59.2% (95% CI: 52.7% 65.5%) of motor riders; it was higher among those with a licence (75%) and those who owned motorbikes (61.4%) (Fig 1).

#### Factors associated with compliance with safety regulations

The univariate analysis shows that age, religion, marital status, motorbike ownership, smoking status and licence were associated with compliance with road safety regulations (Table 3). In the multivariate analysis, increasing age increased the likelihood of compliance with road safety regulations. Motor riders aged between 30–39 and 40–49 years were 2.37 and 4.12 times more likely to comply with road safety regulations compared to those <29 years [AOR: 2.37, 95% CI: 1.07–5.25, p = 0.033] and [AOR: 4.12, 95% CI: 1.36–12.42, p = 0.012], respectively. Motor riders without a licence were 53% less likely to comply with safety regulations than those with a licence [AOR: 0.47, 95% CI: 0.24–0.95, p = 0.037]. Motor-riders who did not own a motorbike were 61% less likely to comply with road safety regulations [AOR: 0.39, 95% CI: 0.19–0.83, p = 0.015] compared to those who owned their motorbikes. Motor riders without alternate occupations were also 49% less likely to comply with road safety regulations compared to those with an alternate occupation aside from riding [AOR: 0.51, 95% CI: 0.27–0.95, p = 0.033] and motorcycle riders who did not smoke were 3.15 times more likely to comply with road safety regulations [AOR: 3.15, 95% CI: 1.47–6.69, p = 0.003] (Table 3).

**Table 3:** Factors associated with compliance with road safety regulation.

Variable	Unadjusted			Adjusted		
	COR	95% CI	p-value	AOR	95% CI	p-value
**Age**						
<29						
30–39	4.35	(2.25–8.41)	<0.001	**2.37**	**1.07–5.25**	**0.033**
40–49	7.36	(2.88–18.78)	<0.001	**4.12**	**1.36–12.42**	**0.012**
**Marital status**						
Married						
Single	0.45	0.25–0.81	0.007	1.17	0.56–2.43	0.663
**Religion**						
Christian						
Muslim	0.56	0.33–0.94	0.031	0.83	0.45–1.54	0.576
**Alternate occupation**						
Present						
Absent	0.42	0.24–0.73	0.002	**0.51**	**0.27–0.95**	**0.033**
**Level of education**						
No education						
Basic education	1.38	0.65–2.91	0.402			
Secondary	2.0	0.81–4.92	0.132			
Tertiary	2.67	0.60–11.80	0.196			
**Area of residence**						
Urban						
Rural	0.98	0.58–1.68	0.948			
**Weekly Income (GHS)**						
<400						
>400	1.38	0.12–15.44	0.793			
**Owner of a motorbike**						
Owns a motorbike						
Does not own a motorbike	0.21	0.11–0.41	<0.001	**0.39**	**0.19–0.83**	**0.015**
**Current smoking status**						
Smokes						
Does not smoke	2.58	1.35–4.91	0.004	**3.15**	**1.47–6.69**	**0.003**
**Current drinking status**						
Drinks						
Does not drink	0.83	0.49–1.41	0.498			
**Ever been involved in a motor accident**						
Ever been involved						
Never been involved	1.54	0.89–2.67	0.121			
**Licensed rider**						
**Licensed**						
Not licensed	0.23	0.13–0.40	<0.001	**0.47**	**0.24–0.95**	**0.037**
**Ever been caught breaking traffic regulations**						
Ever been caught						
Never been caught	1.22	0.70–2.17	0.466			
**Determinant of speed limits**						
Sales						
Competition with fellow riders	0.77	0.37–1.57	0.475			
Concurrent jobs	1.27	0.65–1.57	0.477			

* statistically significant at p-value <0.05: COR-Crude Odds Ratio: AOR-Adjusted Odds Ratio.

## Discussion

The study aimed to assess compliance with road safety regulations among motorcycle riders, and the findings revealed that 59.2% of the respondents complied with road safety regulations. Compliance with road safety regulations is important in the reduction of road traffic fatalities. A study of crash helmet usage in rural Nigeria found a zero-compliance rate [15], which is lower than the compliance rate found in the current study. Apart from helmet usage, another study on traffic regulations compliance with regard to riders’ licence found a 57% compliance rate among commercial motorcyclists [16], which is similar to the results obtained in the present study. The reasons for the high compliance found in the present study could be because a greater proportion of riders scored in the affirmative for the majority of the components used to compute compliance to road safety regulations, including owning and wearing crash helmets, holding a licence, wearing protective clothing when riding and obeying traffic signs. This may have stemmed from personality traits such as agreeableness, which demands cooperation and conscientiousness, which involves the tendency to be organised, disciplined and have a sense of duty. It has been noted that the big-5 personality traits, including agreeableness, conscientiousness, openness, extraversion and neuroticism, are associated with compliance with road traffic regulations [17]. Again, the study revealed that respondents aged between 30–39 years and 40–49 years were 2.37 and 4.12 times, respectively, more likely to comply with road safety regulations. This was affirmed by Olumide and Owoaje [18] in their study, which revealed that younger commercial motorcyclists (less than 25) were associated with low compliance with road safety regulations compared to older and experienced riders. The finding in the current study may be because younger riders had fewer years of practice, which is a proxy for riding experience. Also, the findings in the current study could be because the more a person engages in an activity, the more knowledgeable and accustomed to the rules they become, and hence, the more likely they are to adhere to the rules governing that activity [19]. In this study, motor riders without alternate occupations were 0.51 times less likely to comply with road safety regulations than those with other occupations aside from riding. The reason could be because motor riding is the sole source of income for those without alternate sources of income; thus, the riders may be in a hurry to make some substantial amount of earnings before the close of the day, and this may influence their compliance practices. Also, motor riders who did not own motorbikes were 0.39 times less likely to comply with road safety regulations. This could be compared to findings in a study conducted in Kenya [20], which revealed that the few “boda boda” riders who owned motorcycles may have the capacity to get formal training on compliance to road safety regulations.

Furthermore, they were more likely to be very careful while operating the motorcycle because this was their own source of income, which they used to support their dependents; thus, it ought to be protected. Also, some riders had taken loans to purchase their motorcycle and have to service the loan. It is, therefore, clear that those who did not own motorcycles in this study might not bother about being responsible for servicing and maintaining the motorcycles, which consequently, leads to them mishandling or not complying with safety regulations. Respondents who do not have a licence were 0.47 times less likely to comply with road safety regulations. This is comparable to the findings of a study conducted by Baldi, Baer and Cook in Nigeria [21], which revealed that the low level of compliance was found among motor riders who did not possess a rider’s licence. Owning a rider’s licence starts with receiving training on road safety practices and passing an exam. Hence, it is explainable that those without a licence would be less likely to comply with road safety regulations. In addition, those with riders’ licence regularly have their licence inspected by the police (even though commercial motor riding has not been legalised). Consequently, riders might be forced to obey all road safety regulations in order to avoid sanctions by the police [21].

In this study, those who were not smokers were 3.15 times more likely to comply with road safety regulations. In a similar study conducted in Nigeria, smoking of tobacco and alcohol intake among commercial motor riders had a significant association with previous RTAs [22]. Smoking and alcohol are psychoactive substances that may interfere with a motor rider’s consciousness and judgement while riding. These external factors, including drugs, can interfere with visual acuity, which may contribute to compliance or non-compliance with road safety regulations [23]. It is also important to take note of the engineering implications of roads on compliance with road safety regulations by commercial motorcycle riders. Drivers usually, may be more careful when driving in unfavourable driving environments (intersections and merging ramps) [24] and factors such as vehicle kilometres travelled, air temperature and precipitation may affect crashes with both local and non-local vehicles [25].

Interactivity between slope and visibility, and slope and wind speed [26] have also shown correlation to an upsurge in crash risks. These underscore the roles individual behaviour, road designs and traffic control authorities play in improving compliance with road safety regulations.

## Conclusion

This study reveals 59.2% compliance with road safety regulations among motorcycle riders in the Hohoe township. The findings reveal that age (30 years and above) and having an alternative occupation were the socio-demographic characteristics associated with compliance with road safety regulations. Regarding individual-related factors, those without a rider’s licence and those who did not own their current motorbikes were less likely to comply with road safety regulations. In contrast, those who did not smoke were more likely to comply with road safety regulations. The Ministry of Roads and Highways should liaise with the Drivers and Vehicle License Authority (DVLA) to develop effective road safety compliance educational messages, particularly targeting younger motorcycle riders, those without alternate occupations, those who do not have their own motorbike and those who smoke. This should be done together with routine inspection of riders’ licence by the police, as well as the Motor Transport and Traffic Unit (MTTU), while culprits should be made to face the full rigorous of the law.

Furthermore, attention should be paid to the design and engineering component of roads to improve compliance levels to safety regulations. A major limitation of this study is the potential for reporting bias, as authors could not verify responses to some variables, such as participants’ smoking status and responses to safety regulations compliance. This may translate into under-reporting of certain variables. Also, the all-male respondents in the study could not elicit how female commercial motorcyclist would behave; however, the study highlights important findings to inform stakeholders’ decisions.

## Supporting information

S1 File (DOCX)
