# Supplementary material for: Compliance of commercial motorcycle riders with road safety regulations in a peri-urban town of Ghana
Source: PLoS One. 2021 Mar 30;16(3):e0246965. doi: 10.1371/journal.pone.0246965 (PMC8009399; doi:10.1371/journal.pone.0246965)
Supplement: S1 File — (DOCX) [file pone.0246965.s001.docx]

# **DATA COLLECTION INSTRUMENT (QUESTIONNAIRE**

# **On**

# **Compliance of commercial motorcycle riders to road safety regulations**

You are assured that your response will be kept confidential and used for the research purpose only. ***(You are at liberty to respond to any question of your choice).***

Initials of the interviewer……………….

Date of interview (DD/MM/YY) ………/………/20….

**SECTION A – DEMOGRAPHIC DATA.**

| **1** | Sex of respondent | 1. Male 2. Female |  |
| --- | --- | --- | --- |
| **2** | Age of respondent | (years) |  |
| **3** | Marital status | 1. Married 2. Not married 3. Divorced 4. Widowed |  |
| **4** | Religion | 1. Christian 2. Muslim 3. Traditionalist 4. Other specify……… |  |
| **5** | Do you have an additional job apart from commercial motor riding?  If (“No” to “7”) | 1. Yes [ ] 2. No [ ] |  |
| **6** | Respondent’s additional occupation | 1. Government employed 2. Self-employed 3. Unemployed |  |
| **7** | Respondent’s level of education | 1. Basic education 2. Secondary 3. Tertiary 4. No education |  |
| **8** | Area of Residence | 1. Urban 2. Peri-Urban 3. Rural |  |

**COMPLIANCE TO ROAD SAFETY REGULATIONS**

| 1 | Do you own a crash helmet? | 1. Yes [ ] 2. No [ ] |  |
| --- | --- | --- | --- |
| 2 | Do you wear a crash helmet on your trips? | 1)Always [ ]  2) Sometimes [ ]  3) Don’t like wearing at all [ ] |  |
| 3 | Do you provide a crash helmet for your passengers? | 1. Yes [ ] 2. No [ ] |  |
| 4 | Do you ensure your passengers wear their helmet correctly before you ride? | 1. Yes [ ] 2. No [ ] |  |
| 5 | Do you wear any protective cloth? | 1. Yes [ ] 2. No [ ] |  |
| 6 | What is the usual number of passengers you carry? | 1. One [ ] 2. Two [ ] 3. Three [ ] |  |
| 7 | Do you put on adequate footwear (eg shoes) when riding? | 1. Yes [ ] 2. No [ ] |  |
| 8 | Do you ever smoke? | 1. Yes [ ] 2. No [ ] |  |
| 9 | Have you ever drunk alcohol before?  Do you still drink alcohol?  Do you use alcohol whiles working? | 1. Yes [ ] 2. No [ ] |  |
| 11 | Do you use your headlight on the road? | 1. Yes [ ] 2. No [ ] |  |
| 12 | What is your usual speed limit? | 1. 50km/hr 2. 75km/hr 3. 100km/hr 4. Other specify…………………. |  |
| 13 | What determines the speed you use on the road? | 1. Sales 2. Competition with fellow riders 3. Concurrent jobs |  |
| 14 | How often do your passengers complain when you are riding at maximum speed? | 1. Never/rarely 2. Always 3. sometimes |  |
| 15 | How often do you comply with traffic sign? | 1. Never/rarely 2. Always   sometimes |  |
| 16 | How often do you disregard roads pavement markings? | 1. Never/rarely 2. Always 3. sometimes |  |
| 17 | How often do you wear reflective clothes to when riding? | 1. Never/rarely 2. Always 3. sometimes |  |
| 18 | How often do you wear thick gloves when riding? | 1. Never/rarely 2. Always   3)sometimes |  |
| 19 | How often do you use protective googles when riding? | 1. Never/rarely 2. Always 3. sometimes |  |
| 20 | How often do you stop to rest when tired? | 1. Never/rarely 2. Always 3. sometimes |  |
| 21 | How often do you receive calls when riding? | 1. Never/rarely 2. Always 3. sometimes |  |
| 22 | Do you ride side by side with other riders? | 1. Yes [ ] 2. No [ ] |  |
| 23 | Do you ride with care in areas with pedestrians? | 1. Yes [ ] 2. No [ ] |  |
| 24 | How often do you maintain two space gaps between vehicles | 1. Never/rarely 2. Always 3. sometimes |  |

**SECTION B – INDIVIDUAL RELATED FACTOR ASSOCIATED WITH COMPLIANCE TO ROAD SAFETY REGULATION**

| **1** | Do you have a motorcycle rider’s license? | 1. Yes [ ] 2. No [ ] |  |
| --- | --- | --- | --- |
| **2** | How often do you service your motorbike? | 1. Once in every week 2. Once in every month 3. Once in every year 4. Other (specify)………… |  |
| **3** | Were you involved in a motor accident before as a rider? | `1) Yes [ ]  2) No [ ] |  |
| **4** | How many times have you been involved in a motorbike accident as a rider? | 1. Once 2. Twice 3. 3 times |  |
| **5** | When last were you involved in an accident as a rider? | 1) Month ago  2) 3months ago  3) 6months ago  4) 1year ago |  |
| **6** | Have you received any training on motor riding before working as commercial rider? | 1. Yes [ ] 2. No [ ] |  |
| **7** | If yes to above, who trained you? | 1. Family 2. Friend 3. Training school |  |
| **8** | How often do you renew your license? | 1. 1year 2. 2year 3. 3year |  |
| **9** | Are you the owner of the motor cycle you are using? | 1. Yes [ ] 2. No [ ] |  |
| **10** | What is the average profit you make in a week? | 1. 200cedis 2. 400cedis 3. 500cedis |  |
| **11** | Do you know  the road safety regulations | 1. Yes [ ] 2. No [ ] |  |
| **12** | If yes how did you get to know it | 1. Was taught by highway officials before receiving my license 2. Was taught by colleague rider 3. Watch documentaries on tv |  |
| **13** | Do you show off when riding (over speeding)? how often? | 1. Never/rarely 2. Always 3. sometimes |  |
| **14** | Have you dangerously overtaken any vehicle or motorcycle on the roads? | 1. Yes [ ] 2. No [ ] |  |
| **15** | How often do you overtake them? | 1. Never/rarely 2. Always 3. sometimes |  |
| **16** | Why do you overtake them? | 1)To reach on time  2)To show confidence  3)To stop them from blocking your way |  |
| **17** | Do you compete with other road users on the roads | 1. Yes [ ] 2. No [ ] |  |
|  | What makes you aggressive when riding | 1)when overtaken by other road users  2) when other road user keeps blowing horns on you  Others? |  |
|  | How often do you wrong judgement?  (misjudging distance to change lanes or making tight turns without clearance) | 1. Never/rarely 2. Always 3. sometimes |  |

**SECTION C- SYSTEM RELATED FACTORS ASSOCIATED WITH THE COMPLIANCE TO ROAD SAFETY REGULATIONS**

| 1 | Have you been caught breaking the road safety rules? | 1. Yes [ ] 2. No [ ] |  |
| --- | --- | --- | --- |
| 2 | What happen when you were caught? | 1. got arrested 2. was fined 3. paid bribe and was set free |  |
| 3 | Have you been stopped by the police for over speeding? | 1. Yes [ ] 2. No [ ] |  |
| 4 | If yes how many times? | 1. Once 2. Twice 3. 3times or more |  |
| 5 | What do you do when the roads are blocked by heavy traffic jam? | 1. I use the pedestrian’s way 2. I ride between the heavy traffic 3. I wait patiently till the road is cleared |  |
| 6 | Do heavy traffic jam affect you daily profit? | 1. Yes [ ] 2. No [ ] |  |
| 7 | How often do the highway officials come to educate you on road safety regulations? | 1. Never 2. Once   3)Always |  |
| 8 | Do poor roads affect your ability to use your lanes and control your speed | 1. Yes [ ] 2. No [ ] |  |
| 9 | Do poor weather condition such as flood, windy atmosphere etc. affect your ability to comply to some safety rules. such as over speeding and use of wrong lanes | 1. Yes 2. No |  |
| 10 | How often do the police inspect your riding license? | 1. Never/rarely 2. Always 3. sometimes |  |
